# miR-628, a microRNA that is induced by Toll-like receptor stimulation, regulates porcine innate immune responses

**DOI:** 10.1038/srep12226

**Published:** 2015-07-31

**Authors:** He Jun, He Ying, Chen Daiwen, Yu Bing, Mao Xiangbing, Zheng Ping, Yu Jie, Huang Zhiqing, Luo Junqiu

**Affiliations:** 1 Institute of Animal Nutrition, Sichuan Agricultural University, Ya’an, Sichuan 625014, P. R. China; 2 Key Laborotary of Animal Disease-Resistance Nutrition, Ministry of Education, China

## Abstract

Mammalian innate and acquired immune responses involve a coordinated, sequential, and self limiting sequence of events controlled by positive and negative regulatory mechanism. MicroRNAs have been implicated as a negative regulator for diverse biological events including immune responses. However, the involvement of miRNAs in regulating the immune responses is just beginning to be explored. Here, we characterized the expression profiling of 375 microRNAs in porcine monocytes induced by lipopolysaccharide (LPS), and result shows that several of them are endotoxin-responsive genes. Through promoter analysis, the miR-628 was found to be a NF-κB dependent gene. Importantly, miR-628 was predicted to base-pair with sequences in the 3′-UTR of the myeloid differentiation protein 88 (MyD88) gene. And we found that the UTR inhibit expression of a linked reporter gene coding a key adapter molecule downstream of Toll-like receptors (TLRs), resulting in suppressing of the TLR signaling. Therefore, we not only propose a role of miR-628 in control of the TLR signaling through a negative feedback regulation loop involving down-regulation of MyD88 protein levels, but results may also contribute to rational target selection orchestrating the inflammatory responses.

The innate immune response of mammals offers a pivotal first line of defense against diverse pathogens. Activation of the elements of innate immunity results in release of cytokines and chemokines. The interleukins (ILs), along with tumor necrosis factor-alpha (TNFα) and chemokines help regulate inflammation, the intensity of immune response, and play a role in activating the adaptive immune response^1^. Monocytes are central cells of the innate immune system that is essential in the initial host reaction to infection by initiating an inflammatory response (i.e. release of inflammatory cytokines). Activation of monocytes is triggered by the recognition of self and non-self stimuli mediated through a myriad of specialized membrane and intracellular receptors^2^,^3^.

The toll-like receptors (TLRs) are a class of integral memebrane glycoproteins containing an extracellular domain with leucine-rich repeat motifs and a cytoplasmic domain responsible for “self”-signal recognition^4^,^5^. In mammalian monocytes and macrophages TLRs 1, 2, 4, 5, 6 and 10 have been identified in the plasma membrane. Intracellularly, TLRs 3, 7 and 9 are found in the endosome membranes, whereas only TLR9 is found in the endosome membranes in monocytes^5^. All TLRs triggers signals in a similar fashion because of the presence of Toll and IL-1 receptor (TIR) domain in their cytoplasmic tails. Following activation, TLRs recruit adaptor molecules within the cytoplasm of cells to propagate a signal, which ultimately leads to the induction or suppression of genes that orchestrate the inflammatory response^6^,^7^. Currently, four adaptor molecules, namely MyD88, Tirap, Trif, and Tram were identified in the TLR signaling^8^,^9^,^10^. It is noteworthy that TLR4 is the only TLR that is capable of recruiting all the four adaptors, and is activated by diverse pathogen-associated molecular patterns (PAMPs) including LPS from Gram-negative bacteria, fusion (F) protein from respiratory syncytial virus and the evelope protein from mouse mammary rumor virus^11^,^12^,^13^.

LPS is one of the best studied immunostimulatory components of bacteria that can induce systemic inflammation and sepsis if excessive signals occur^14^. Previous study showed that the LPS-initiated signaling cascade of TLR4 was transduced through MyD88-dependent and MyD88-independent pathways^5^. Following LPS stimulation, MyD88 activates two death domain-containing kinases, IL-1 receptor associated kinase-4 (IRAK4) and IRAK1 successively, and then recruits into the complex TNF receptor-associated factor 6 (TRAF6). This chain of events triggers activation of IκB kinase and JNK and, in turn, the downstream of NF-κB and AP-1 transcriptional factors resulting in up-regulation of immune-responsive genes (i.e. proinflammatory cytokines)^15^,^16^. The MyD88-independent branch (i.e. TRIF) of signaling leads to the activation of another group of transcriptional factors and results in a boost expression of IFNs and other genes that are important for anti-viral and anti-bacteria responses^17^. Although, activation of TLR4 is important for host to help fighting infections, these responses can be detrimental if they are excessively prolonged or intense (i.e. acute sepsis)^18^. Therefore, inhibitory pathways are necessary to protect the host from inflammation-induced damage.

MicroRNAs (miRNAs) are 21–22-nucleotide, non-coding small RNAs that have been identified as a negative regulator for diverse biological events and impact protein expression at the translational level^19^. Recent studies has indicated that a range of miRNAs are involved in the regulation of immunity, including the development and differentiation of B and T cells (miR-17 ~ 92), proliferation of monocytes and neutrophils (miR-20a, miR-155, and miR-233), antibody switching and the release of inflammatory mediators (miR-146a)^20^. In addition, miRNA expression is also impacted by immune mediators in some model systems. For instance, LPS impacts expression of miR-9 and miR-146 in human THP-1 monocytes^21^,^22^. While both IFN-α and INF-β can modulate expression of several miRNAs required for their anti-viral responses following infection with hepatitis C virus^23^. As the miRNAs are usually considered to be a negative regulator controlling the immune response, it is very important to further explore the ability of inflammatory ligands to modulate miRNA expression, and the role of regulated miRNAs in the development of an adequate immune response is also need to be further investigated.

In the present study, we characterized the expression profiling of 375 miRNAs in porcine monocytes exposed to LPS. We show that several miRNAs (i.e. miR-628) are acutely induced. Promoter analysis of the *miR-628* gene indicated that NF-κB plays a critical role in induction of its transcription by LPS. In addition, the MyD88 gene was firstly identified as a potential molecular target of miR-628 through experiments with 3′ UTR luciferase reporters. These findings not only probe a novel mechanism maintaining the balance of TLR signaling, but may also help select rational targets fine-tuning the inflammatory responses.

## Results

### miR-628 is an immediate early-response gene induced by LPS

**Figure 1:**
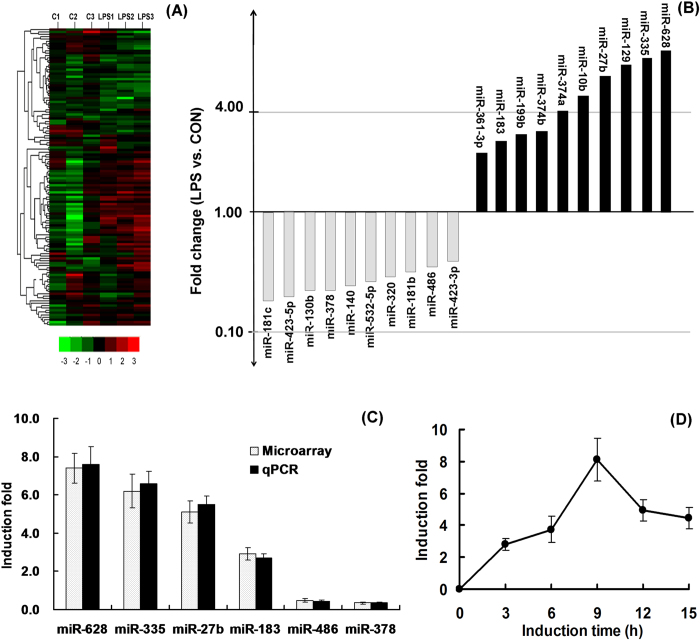
Expression profiling of mature miRNAs in porcine monocytes following LPS stimulation. (**A**) Heat-map of the miRNAs expressed in porcine monocytes following LPS stimulation. (**B**) Typical examples of selected miRNAs that showed significant changes following LPS stimulation. (**C**) qPCR validation of selected miRNAs. (**D**) Kinetics of miR-628 expression following LPS stimulation. Porcine monocytes were stimulated by LPS for the indicated times, and miR-628 expression was analyzed by qPCR. (* means P<0.05; **means P<0.01).

To globally assess the miRNAs that are potentially involved in the responses of peripheral immune cells to stimuli of bacterial origin, the miRNA expression profiling was investigated in porcine monocytes simulated for 9 h with 100 ng/mL LPS using a TaqMan-based Low Density Array. Result shows that the expression pattern of miRNAs in porcine monocytes was significantly affected by LPS stimulation, and 25 miRNAs were observed to be significantly up-regulated and 15 miRNAs were down-regulated in LPS-treated cells (*P* < 0.05). Typical examples of selected miRNAs that showed significant changes (ten of the most up-regulated and down-regulated miRNAs) are shown in Fig. 1B. LPS-induced miRNAs identified in the array were further validated by quantitative RT-PCR (qPCR) analysis. Consistent with the array data, the expression levels of miR-628, miR-335, miR-27b, and miR183 significantly increased in LPS-treated monocytes, while the expression of miR-486 and miR-378 acutely decreased (Fig. 1C). The miR-628 was chosen for detailed analysis in the present study, since it shows a maximal increase following LPS stimulation (about 8-fold) and remains unknown in the regulation of inflammatory responses. The rapid induction of miR-628 by LPS, as shown by qPCR, suggested that miR-628 might be an early-response gene against LPS stimulation. Its expression has reached a peak value after 9 h induction. (Fig. 1D).

### miR-628 is an NF-κB dependent gene

**Figure 2:**
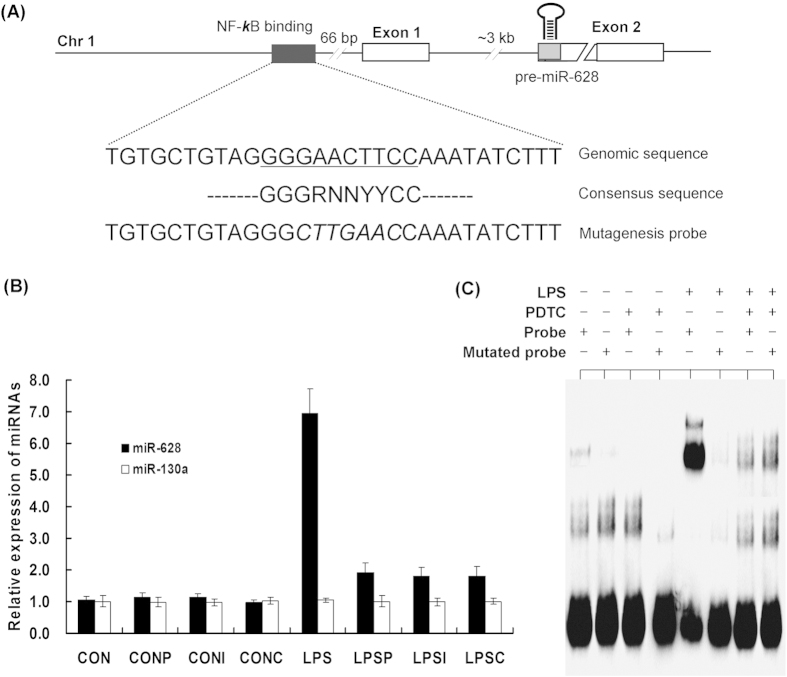
LPS-induced expression of miR-628 gene is NF-κB-denpendent. (**A**) Schematic diagrams of miR-628 genomic loci on porcine chromosomes 1. Putative binding sites of NF-κB transcriptional factors are shown as black boxes. (**B**) Effect of NF-κB inhibitors on miR-628 expression in porcine monocytes (PDTC, 100 μM; IL-10, 10 ng/ml; Curcumin, 50 μg/ml). CON, non-stimulated cells; CONP, CON + PDTC; CONI, CON + IL-10; CONC, CON + Curcumin; LPS, LPS-stimulated cells; LPSP, LPS + PDTC; LPSI, LPS + IL-10; LPSC, LPS + Curcumin. (**C**) Electrophoretic mobility shift assays. Lanes 1–8, reaction mixtures containing nuclear extract (15 μg) and probe with additions as indicated. (* means P<0.05; **means P<0.01).

Stimulation of mammalian monocytes with LPS results in transcription of a variety of NF-κB-dependent genes controlling cellular growth, differentiation, survival and apoptosis^24^,^25^. To investigate whether activation of the TLR/NF-κB signaling is involved in the transcription of miR-628, we performed a promoter analysis for this miRNA by using database search (http://asia.ensembl.org/index.html). We found that the porcine miR-628 gene is located on chromosome 1, and analysis of the data shows that the gene consists of two exons separated by ≈3 kb of genomic sequence (Fig. 2A). A putative NF-κB binding site (*GGGRNNYYCC*) was found to be located 66 bp upstream of the exon1.

To investigate the role of TLR/NF-κB activating in pre-miR-628 expression, we performed qPCR analysis of levels of miR-628 expression in LPS-stimulated monocytes using a specific NF-κB inhibitor Pyrrolidine dithiocarbamate (PDTC) and two conventional immune suppressants (Curcumin and IL-10). We found that the miR-628 was rapidly induced after LPS-stimulation (Fig. 2B). However, the stimulatory effect of LPS was not only significantly suppressed by PDTC, but also suppressed by Curcumin and IL-10 (*P* < 0.01). The miR-130a was used as a control, since it is not NF-kB-dependent (result from microarray analysis). As expected, the expression level of miR-130a was not affected by LPS or NF-κB inhibitors (*P* > 0.05). Coupled with the putative NF-κB binding site, these results suggested that the miR-628 induction by LPS may result from the transcriptional activity of NF-κB.

We next validated the putative NF-κB binding site by Electrophoretic Mobility Shift Assay (EMSA) using a LightShift Chemiluminescent EMSA Assay Kit. A biotin-labeled probe which covers the putative binding sequence was used to determine whether a protein bound to this region. As shown in Fig. 2C, the probe bound to nuclear extracts from LPS-stimulated cells, whereas a mutant probe (mutation of 6 nt in the putative binding sequence) bound only few amount of protein from the nuclear extracts. Moreover, treatment the cells with a specific NF-κB inhibitor (PDTC) has resulted in significant reduction of the binding. These results indicated that the LPS-induced expression of *miR-628* gene is NF-κB-denpendent.

### Targeting of MyD88 mRNA by miRNA-628

**Figure 3:**
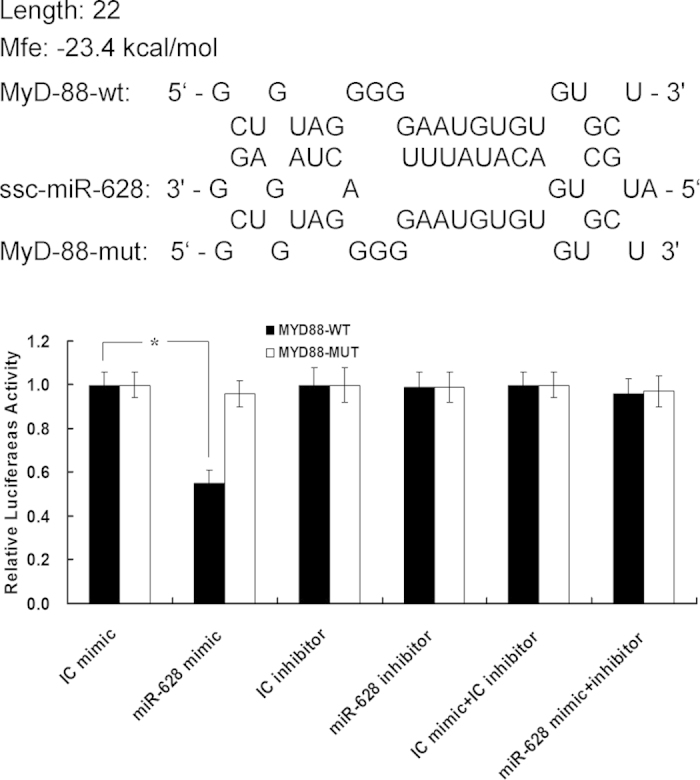
MyD88 is a target of miR-628 posttranscriptional repression. Shown is a sequence alignment of miR-628 and its target sites in 3′UTR of MyD88. Also shown is an analysis of expression of MyD88-UTR luciferase reporters in the presence of miR-628/inhibitor or the irrelevant control (IC). Filled bars correspond to reporter constructs with wild-type miR-628 targeting sites, and open bars correspond to constructs with 6-nt substitutions disrupting base-pairing with the “seed region” of miR-628. (* means P<0.05; **means P<0.01).

It has been shown that miRNAs not only target mRNA for degradation but also can inhibit mRNA translation^26^. To explore the functional consequences of miR-628 induction by LPS, we searched for predicted mRNA target sites using TargetScan (http://www.targetscan.org). We found that 3′ UTRs of the gene coding MyD88 protein contains a potential miR-628 target sequence (Fig. 3). MyD88 is a key adapter molecule in TLR and IL-1 receptor signaling cascades, mediating activation of NF-κB and AP-1 pathway^27^.

To determine whether the predicted target sites are targeted by miR-628, we generated reporter constructs that contain the firefly luciferase gene fused to ≈500 bp of the 3′ UTR from MyD88 mRNA containing putative miR-628 target site. We co-transfected those reporter constructs into HEK-293 cells with a Cytomegaovirus (CMV) promoter-driven β-galactosidase expression plasmid and normalized luciferase activity for transfection efficiency to β-galactosidase activity. We also normalized the luciferase activity obtained after transfection of the miRNA mimic for miR-628 to that obtained with a co-transfected control mimic miRNA with no sequence specificity. As shown in Fig. 3, the mimic miRNA has resulted in lower luciferase activity produced by the wild-type 3′ UTR reporter construct than by the mutant construct (*P* < 0.05). However, the inhibitory effect of mimic miRNA was abrogated by a specific miRNA inhibitor. These results suggested that the MyD88 gene is a target for posttranscriptional repression by miR-628.

### MyD88-targeting miRNA affects the TLR signaling

**Figure 4:**
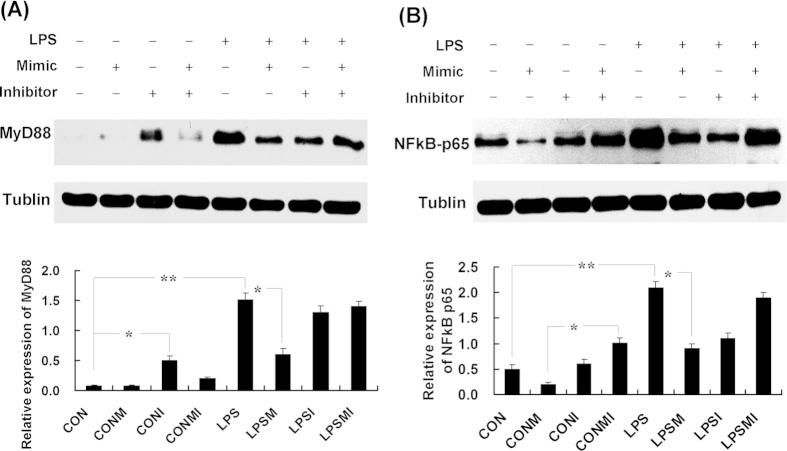
MyD88-targeting miRNA affects the TLR signaling. (**A**) Western-blot analysis of MyD88 protein in porcine monocytes. (**B**) Western-blot analysis of nucleus NF-κB protein in porcine monocytes. CON, non-stimulated cells; CONM, CON + mimics; CONI, CON + inhibitors; CONMI, CON + mimics + inhibitors; LPS, LPS-stimulated cells; LPSM, LPS+ mimics; LPSI, LPS- + inhibitors; LPSMI, LPS + mimics + inhibitors. (* means P<0.05; **means P<0.01).

**Figure 5:**
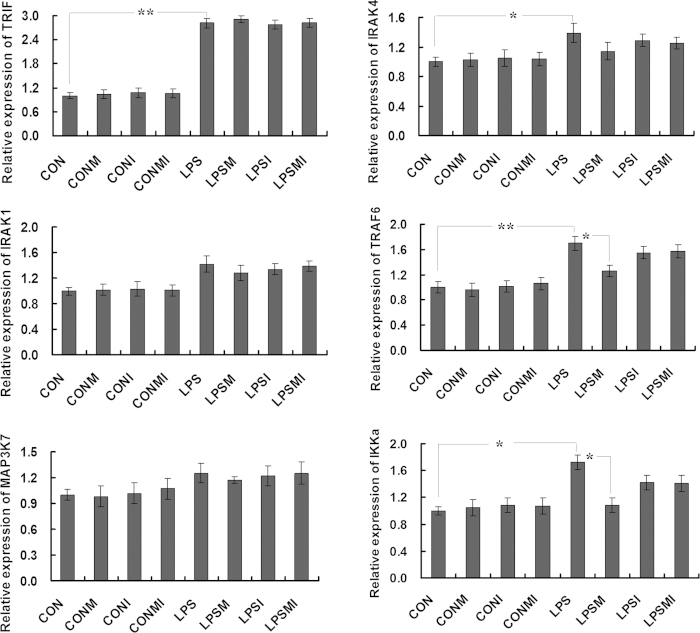
qPCR analysis of selected adapters of the TLR signaling. CON, non-stimulated cells; CONM, CON + mimics; CONI, CON + inhibitors; CONMI, CON + mimics + inhibitors; LPS, LPS-stimulated cells; LPSM, LPS+ mimics; LPSI, LPS- + inhibitors; LPSMI, LPS + mimics + inhibitors. (* means P<0.05; **means P<0.01).

As MyD88 is a key adapter molecule in TLR signaling, we next investigated the influences of miR-628 on expression levels of MyD88 and its downstream components. Porcine monocytes transfected with miR-628 mimic or inhibitors were incubated with 100 ng/ml LPS for 9 h. The transcfection efficiency was confirmed by detection of the miR-628 expression using qPCR (Supplementary file1). A NE-PER Reagent Kit (Thermo Scientific) was used to isolate the cytoplasmic and nuclear extracts. As expected, the protein level of MyD88 significantly increased upon LPS stimulation (*P* < 0.01). However, it was significantly down-regulated by the miR-628 (*P* < 0.05). Transfection of the cells with a specific miRNA inhibitor has resulted in abortion of the inhibitory effect (Fig. 4A). It is a well known fact that NF-κB is the most important downstream effector of TLR4 signaling, triggering the transcription of genes that orchestrate the inflammatory response^6^,^11^. We found that the protein level of nuclear NF-κB p65 was significantly elevated in LPS-stimulated cells, whereas transfection the cells with miR-628 has resulted in a down-regulation of this protein (Fig. 4B). Moreover, we investigated the expression levels of other adapter molecules downstream of MyD88 by using qPCR (Fig. 5). Interestingly, we observed an elevated expression of several adapters (TARF6 and IKKα) in LPS-stimulated cells (*P* < 0.05). However, the expression levels of TARF6 and IKKα was down-regulated by the miR-628 (Fig. 4C). It is noteworthy that the expression level of TRIF (a key LPS-inducible adapter involved in the MyD88-independent pathway) was not affected by the miR-628 transfection (*P* > 0.05). These findings indicated a negative regulatory function of miR-628 controlling the MyD88-dependent TLR signaling.

## Discussion

TLR activation is a double-edged sword. It is essential for initiating the innate response and regulating adaptive immunity against pathogens^4^,^28^. However, over-activation of the TLR family (or induced too strongly) can be detrimental, since it has been implicated in the pathogenesis of autoimmune, chronic inflammatory and infectious disease (i.e. sepsis)^29^. Therefore, the immune system needs to constantly strike a balance between activation and inhibition to avoid detrimental and inappropriate inflammatory responses, and the TLR signaling must be tightly regulated. Currently, miRNAs has been characterized as a negative regulator for many biological events, including the immune responses. A rapid increase in the expression of several selected miRNAs was observed during the activation of an innate immune response^22^. However, the relevance of miRNAs in this process is just beginning to be explored.

In the present study, we investigated the potential involvement of miRNAs in the regulation of innate immune response by characterizing the profile of miRNAs expressed in porcine monocytes following LPS induction, and significant alterations in miRNA expression profiles were observed. In addition to previous identified miRNAs (i.e. miR-9 and miR-146) induced by LPS in mouse or human monocytes^21^,^22^, a new set of miRNAs (miR-628, miR-335, and miR-27b) were found to be LPS-responsive miRNAs in porcine monocytes. The miRNA expression profile was also different from previous reports in which the expression of miRNAs was explored in pigs infected with diverse bacteria^30^,^31^. Among these identified miRNAs, the expression level of *miR-628* gene was found to be enhanced considerably. But the role of miR-628 in the regulation of the inflammatory responses is still unknown. In the present study, an RT-qPCR-based screening on LPS-treated monocytes not only confirmed the induction of miR-628, but also suggested that the *miR-628* gene is potentially transactivated via promoter binding of the NF-κB, since the LPS is a well-known immunostimulatory component activating the TLR signaling^15^,^16^.

The NF-κB family of transcription factors consists of five members, p50, p52, p65 (RelA), c-Rel, and RelB. However, the transcription activation domain (TAD) necessary for the positive regulation of gene expression is present only in p65, c-Rel, and RelB^32^. Increased nuclear translocation of p65 and p50 was previously observed in mouse and human macrophages following LPS stimulation^33^,^34^. Through promoter analysis, we identified a putative NF-κB binding site locating upstream of the *miR-628* gene. And result from EMSA clearly indicated that the LPS-mediated elevation of miR-628 expression occurs in an NF-κB-dependent manner.

The results from computational miRNA target prediction algorithms include more potential targets than we tested, but the MyD88 gene yield particularly high scores. Previous study has indicated that mammalian miRNAs may be involved in regulation of multiple independent physiological processes through targeting a wide spectrum of gene targets^22^,^35^. MyD88 is the most critical downstream adaptor for most Toll-like receptors, and inhibition of MyD88 causes abrogation of NF-κB activation^36^. Cotransfection experiments of miR-628 with 3′UTR luciferase reporters identified MyD88 as miR-628 target. The relevance of miR-628 in the regulation of inflammatory responses was further investigated, and significant down-regulation of levels of MyD88 and NF-κB p65 proteins was observed in monocytes transfected with miR-628. We also investigated the expression levels of several adapters downstream of MyD88 by qPCR. Interestingly, the expression level of TARF6 and IKKα was also reduced. This is probably due to the down-regulation of MyD88 after miR-628 transfection^5^. Thus this miRNA could be added to the list of potential negative regulators of inflammation, and we can propose a novel feedback regulation loop, namely bacterial components (i.e. LPS) activate NF-κB, resulting in up-regulation of the miR-628 gene, which, in turns, down-regulate level of MyD88 protein and suppress the activity of this signaling pathway. Additionally, our result presents new evidence that 3′UTR of a single mRNA may be target of multiple miRNAs, since the *MyD88* gene has also been identified as a target of miR-155^37^. Two miRNAs acting together may bring about better repression of the target gene. But as there are many miRNAs for a specific gene, knowledge of most of them will be necessary to elucidate its actual effect on its target gene^38^.

In summary, this first miRNA profiling in porcine monocytes in response to LPS induction indicated significant alterations in cellular miRNA expression. The mechanism by which LPS induces up-regulation of miR-628 in porcine monocytes involves transactivation of miRNA genes through promoter binding of the NF-κB. In addition, the miR-628 inhibits LPS-activated TLR signaling by targeting the MyD88. These data demonstrate a critical role for miRNAs in inflammatory responses and may provide new insights into general mechanisms of maintaining the balance of TLR signaling. Moreover, the results may also contribute to rational target selection for therapeutic interventions preventing the inflammatory responses from becoming detrimental to the host.

## Methods

### Cell purification and culture

Porcine monocytes were separated by centrifugation on Ficoll-Paque Plus (GE-Healthcare) under endotoxin-free conditions using anticoagulated blood from healthy donors (weaned pigs). Monocytes (>99.8% pure) were obtained by non-magnetic selection performed with a CD14 M-pluriBead^®^ kit (pluriSelect). The purity of cell populations was assessed by flow cytometry. Monocytes (2 × 10^6^/ml) were then resuspended RPMI medium 1640 (Gibco) supplemented with 10% FCS (Gibco), 100 units/ml penicillin, 100 units/ml streptomycin, and 2 mM L-glutammine (Sigma). For luciferase reporter assays, HEK293 cell line was grown in DMEM (Gibco) supplemented with 10% FCS, 100 units/ml penicillin, 100 units/ml streptomycin, and 2 mM L-glutamine. Cells were cultured in a humidified incubator with 5% CO_2_ at 37 °C.

### RNA isolation and quantification of miRNAs expression level

Monocytes were stimulated with 100 ng/ml LPS for 9 h and the RNA fraction that is highly enriched for small RNA species was isolated by using the mirVana isolation kit (Ambion), according to the manufacturer’s protocol. The RNA fraction (fifty microgram) was labeled with either Cy3 (control samples) or Cy5 (LPS-treated samples) fluorescent dyes (Amersham Pharmacia). The labeled samples were next mixed and hybridized for 14 h with miRNA array slides. Microarrays were prepared by robotic spotting of DNA oligonucleotide probes complementary to 375 miR sequences (miRNA Probe Set V15, Capitalbio) on epoxy-coated slides (Agilent) in quadruplicate. After hybridization, microarrays were washed as recommended by the manufacturer and scanned by using the GenePix 4200 fluorescent scanner (Axon).

### Real time RT-PCR (RT-qPCR)

For real-time PCR analysis of mature miRNAs, an amount of 50 ng total RNAs was reverse-transcribed by using the Taqman MicroRNA Reverse Transcription Kit (Ambion). Comparative real-time PCR was performed in triplicate using Taqman Universal PCR Master Mix (Ambion) on the Applied Biosystems 7500 FAST real-time PCR system. Mature miRNA-specific primers and probes were obtained from Riobio Inc (Riobio Inc, Guangzhou). Normalization was performed by using RNU6B primers and probes. Relative expression was calculated by using the comparative CT method^39^. For real-time PCR analysis of adapter molecules, total RNAs was reverse-transcribed by using a two-step RT-PCR kit (Takara), and normalization was performed by using GAPDH.

### Electrophoretic mobility shift assay (EMSA)

EMSA was performed by using a LightShift Chemiluminescent EMSA Assay Kit (Thermo Scientific). A biotin-labeled probe containing the predicted NF-κB binding sites upstream of the miR-628 were obtained from Takara (Takara Bio, Dalian). For sample preparation, the monocytes were (2 × 10^6^/ml) stimulated with or without 100 ng/ml LPS for 9 h and the nuclear protein was extracted by using a NE-PER Reagent Kit (Thermo Scientific). Briefly, nuclear extracts were incubated with probes at room temperature for 20 minutes, and loading buffer was added to each after the binding reaction was complete. The reactions were loaded onto a pre-run gel (the native polyacrylamide gel in 0.5 × TBE was pre-run at 100 V for 35 min), and the gel was then run until the bromophenol blue dye migrates to the bottom of the gel. Once complete, the proteins from the gel were transferred to a nylon membrane, and DNA is crosslinked to the membrane for 15 min using a transilluminator (Venoscope) with 312 nm bulb. The biotin-labeled probe was detected by chemiluminescence following the protocol and using the buffers provided in the kit.

### 3′-UTR luciferase reporter assays

To create 3′ UTR luciferase reporter constructs, fragments (about 500 bp) of 3′ UTRs of MyD88 genes was cloned downstream of CMV-driven firefly luciferase cassette in pMIR-REPORT vector (Ambion). Mutated constructs carrying 4-bp substitutions in the miR-628 target sites, were obtained by site-directed mutagenesis. For miRNA target validation assays, 1 × 10^5^ HEK293 cells in a 24-well dish were transiently transfected with 10 ng of each firefly luciferase reporter plasmids, 10 ng pCSK-LacZ vector (for normalization), and 150 ng of miR-628 mimics or inhibitors using Lipofectamine 2000 (Invitrogen) according to the manufacturer’s protocol. The transfection experiment was performed in triplicate. After 48 h cells were lysed and the luciferase activities were determined by using a GoMax 96 Microplate Luminometer (Promega). The values of firefly luciferase activity were normalized by β-galactosidase activities. The normalized values were expressed as changes relative to the value of negative control, which was set as 1.

### Western blot analysis

Cells were treated as described in Results and expression of proteins was analyzed by immunoblotting for MyD88 and NF-κB. Briefly, the protein extracts (50 μg) were separated by SDS-PAGE after adding 5 × Laemmli sample buffer and boiling (For statistics, the gels were run in triplicate). The separated proteins were blotted on to a PVDF membrane and probed with a rabbit polyclonal antibody against amino acids at the C-terminus of MyD88 and NF-κB of porcine origin (Santa Cruz Biotechnol, Inc.). The goat anti-rabbit IgG-HRP (Santa Cruz Biotechnol, Inc.) were used as the secondary antibody. Bound antibodies were detected by the ECL Prime western blotting detection reagent (GE-Healthcare) using a CCD-based imager (ImageQuant LAS 4000).

### Statistical analysis

Data are presented as means ± SD and were analyzed by two-sided non-parametric Student’s *t*-test, unless otherwise indicated. For analysis of more than two groups, a one-way analysis of variance (ANOVA) was used. A P-value of 0.05 or less was considered significant.

## Additional Information

**How to cite this article**: Jun, H. *et al.* miR-628, a microRNA that is induced by Toll-like receptor stimulation, regulates porcine innate immune responses. *Sci. Rep.* **5**, 12226; doi: 10.1038/srep12226 (2015).

## Supplementary Material

Supplementary Information
