# Supplementary material for: miR-628, a microRNA that is induced by Toll-like receptor stimulation, regulates porcine innate immune responses
Source: Sci Rep. 2015 Jul 31;5:12226. doi: 10.1038/srep12226 (PMC4521185; doi:10.1038/srep12226)

miR-628, a microRNA that is induced by Toll-like receptor stimulation, regulates porcine innate immune responses

He Jun[1,2*], He Ying[1,2#], Chen Daiwen[1,2*] , Yu Bing[1,2], Mao Xiangbing[1,2], Zheng Ping[1,2], Yu Jie[1,2], Huang Zhiqing[1,2], Luo Junqiu[1,2],

[1]Institute of Animal Nutrition, Sichuan Agricultural University, Ya'an, Sichuan 625014, P. R. China

[2]Key Laborotary of Animal Disease-Resistance Nutrition, Ministry of Education, China

Supplementary information file

Supplementary file1. qPCR was performed to detect the transfection efficiency of miR-628 mimic and inhibitor in porcine monocytes. At 24 h after transfection, miR-628 expression was up-regulated in mimic group and down-regulated in inhibitor group as compared to the control.

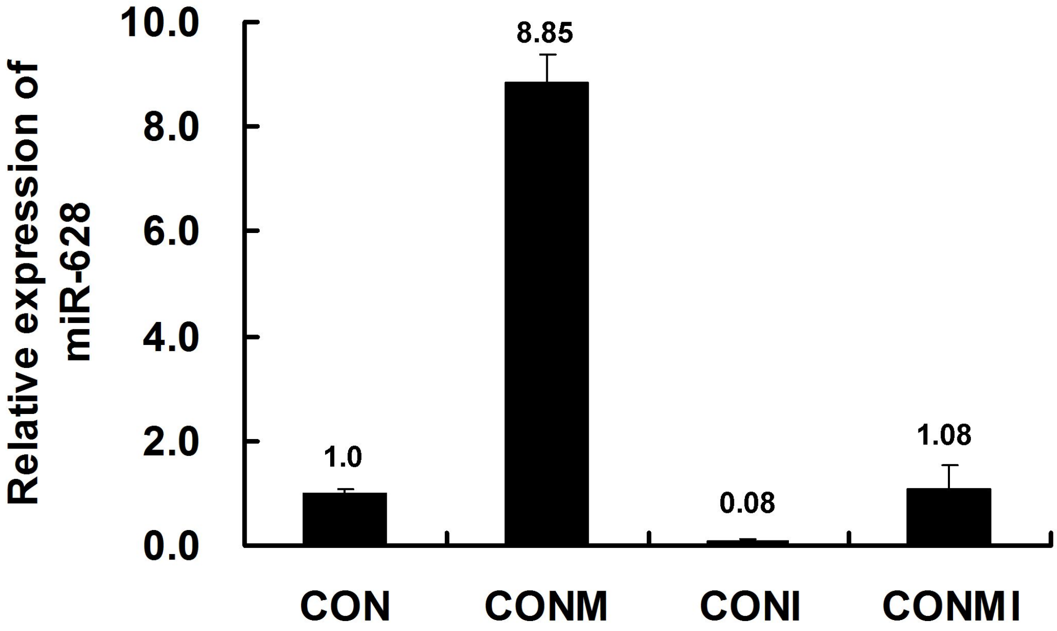

Supplement: Supplementary Information [file srep12226-s1.pdf]
